# Green Synthesized Silver Nanoparticles Immobilized on Activated Carbon Nanoparticles: Antibacterial Activity Enhancement Study and Its Application on Textiles Fabrics

**DOI:** 10.3390/molecules26133790

**Published:** 2021-06-22

**Authors:** Pratama Jujur Wibawa, Muhammad Nur, Mukhammad Asy’ari, Wijanarka Wijanarka, Heru Susanto, Heri Sutanto, Hadi Nur

**Affiliations:** 1 Department of Chemistry, Faculty of Sciences and Mathematics, Diponegoro University, Jalan Prof. H. Soedarto, SH. No.1 Tembalang, Semarang 50275, Indonesia; asyari@live.undip.ac.id; 2 Department of Physics, Faculty of Sciences and Mathematics, Diponegoro University, Jalan Prof. H. Soedarto, SH. No.1 Tembalang, Semarang 50275, Indonesia; mnur@lecturer.undip.ac.id (M.N.); herisutanto@live.undip.ac.id (H.S.); 3 Department of Biology, Faculty of Sciences and Mathematics, Diponegoro University, Jalan Prof. H. Soedarto, SH. No.1 Tembalang, Semarang 50275, Indonesia; Wijanarka@lecturer.undip.ac.id; 4 Department of Chemical Engineering, Faculty of Enginering, Diponegoro University, Jalan Prof. H. Soedarto, SH. No.1 Tembalang, Semarang 50275, Indonesia; herususanto@lecturer.undip.ac.id; 5 Center for Sustainable Nanomaterials, Inbu Sina Institute for Scientific and Industrial Research, Universiti Teknologi Malaysia, Skudai 81310, Johor, Malaysia; hadinur@utm.my; 6 Central Laboratory of Minerals and Advanced Materials, Faculty of Mathematica and Natural Sciences, State University of Malang, Malang 65145, Indonesia

**Keywords:** antibacterial activity enhancement, activated carbon nanoparticle, silver nanoparticle, textile fabrics, *E. coli*, *S. aureus*

## Abstract

This research aimed to enhance the antibacterial activity of silver nanoparticles (AgNPs) synthesized from silver nitrate (AgNO_3_) using aloe vera extract. It was performed by means of incorporating AgNPs on an activated carbon nanoparticle (ACNPs) under ultrasonic agitation (40 kHz, 2 × 50 watt) for 30 min in an aqueous colloidal medium. The successful AgNPs synthesis was clarified with both Ultraviolet-Visible (UV-Vis) and Fourier Transform Infrared (FTIR) spectrophotometers. The successful AgNPs–ACNPs incorporation and its particle size analysis was performed using Transmission Electron Microscope (TEM). The brown color suspension generation and UV-Vis’s spectra maximum wavelength at around 480 nm confirmed the existence of AgNPs. The particle sizes of the produced AgNPs were about 5 to 10 nm in the majority number, which collectively surrounded the aloe vera extract secondary metabolites formed core-shell like nanostructure of 8.20 ± 2.05 nm in average size, while ACNPs themselves were about 20.10 ± 1.52 nm in average size formed particles cluster, and 48.00 ± 8.37 nm in average size as stacking of other particles. The antibacterial activity of the synthesized AgNPs and AgNPs-immobilized ACNPs was 57.58% and 63.64%, respectively (for *E. coli*); 61.25%, and 93.49%, respectively (for *S. aureus*). In addition, when the AgNPs-immobilized ACNPs material was coated on the cotton and polyester fabrics, the antibacterial activity of the materials changed, becoming 19.23% (cotton; *E. coli*), 31.73% (polyester; *E. coli*), 13.36% (cotton; *S. aureus*), 21.15% (polyester; *S. aureus*).

## 1. Introduction

It has been widely known that silver nanoparticles (AgNPs) have functional antibacterial properties towards wide spectrum ones from Gram-positive up to Gram-negative bacteria with low risk for human health [1,2,3]. Many researchers have used AgNPs for a long time as antibacterial agents in various industrials products such as cosmetics [4,5], food storage, packaging [6,7], health devices industry [8,9], textile coatings [10,11], etc. On the other hand, it has been also widely known that both pathogenic bacteria of *Bacillus subtilis* (*B. subtilis*), and *Escherichia coli* (*E. coli*) as the representatives of Gram-positive and Gram-negative bacteria, respectively, are commonly present ubiquitously in various natural environment bodies such as soil, wastewater, and air [12,13]. Due to these media frequently being in contact with humans, the bacteria can reach the human body and subsequently generate many infectious and various diseases. To avoid this dangerous threat, one can use an enhanced high effective antibacterial agent of silver nanoparticles (AgNPs) via preemptive strategy using activated carbon nanoparticles (ACNPs) coated on human clothing materials such as cotton and polyester fabrics.

Unfortunately, almost all the proposed antibacterial mechanisms of AgNPs releasing reactive oxygen species (ROS) radicals [14,15,16] can damage the human cell and induce serious degenerative diseases [17]. To minimize these risks, it is compulsory to use a suitable radical scavenger in using AgNPs as the antibacterial agent in many various applications. In conjunction with that, metal oxide particles such as Aluminum oxide (Al_2_O_3_), Cerium oxide (CeO_2_), and Yttrium oxide (Y_2_O_3_) could be applied for the ROS catching [14]. However, metal oxides are commonly expensive in price, not environmentally friendly, and not easy to produce. Therefore, utilization of other high potential ROS adsorbers that are cheaper in price, renewable in sources, sustainable in their availability, safer for humans, and environmental benign in properties, is a must. In this context, activated carbon nanoparticles (ACNPs) have become the best alternative material, fulfilling all the requirements for the ROS adsorber.

As is widely known, ACNPs can be produced quickly from any biomass of plantation, agriculture, and forestry wastes [18,19,20,21], even from industrial livestock wastes [22]. This situation provides remarkable contribution in reducing severe environmental damage. In addition, the high porosity of the ACNPs with various basins in nano-scale size and depths formed on its surface [18] make the ACNPs the most promising material to skip out the ROS all at once to enhance the antibacterial activity of the AgNPs through the synergistic effect mechanism. The possibility of the ROS adsorption process would happen on the surface of the ACNPs, supported by the facts that activated carbon-based materials have been used to adsorb carbon dioxide (CO_2_) [23], hydrogen peroxide (H_2_O_2_) [24], hydrogen (H_2_) [25], and environmental benign organic molecules [26]. The relationship between surface morphology and potential adsorption of the activated carbon-based materials has been reviewed in detail [27,28,29,30]. This paper comprehensively describes the results of the investigation of the role of ACNPs in enhancing the antibacterial activity of AgNPs towards *E. coli* and *S. aureus* bacteria, especially when it was superimposed onto the cotton and polyester fabrics.

## 2. Experimental Section

### 2.1. Materials

This research used local commercially available carbon black fine powder; Silver nitrate (AgNO_3_) p.a; Distilled water; Phosphoric acid (H_3_PO_4_) p.a; Sodium hydroxide (NaOH) p.a. Agarose powder; Yeast extract; Peptone; Sodium chloride (NaCl) p.a; Amoxicillin, p.a.; Whatman filter paper No.41. Sigma-Aldrich, Germany, produced all the chemicals.

### 2.2. Equipment

Commonly used laboratory glassware; Autoclave sterilizer (Laboratory specification, Linden, Germany); Oven (Cosmos CO-9919, North Jakarta, Indonesia), Furnace (Vulcan 3-130, USA), Ultrasonic cleaner (Krisbow CD4862, Shenzhen, China), pH meter (Senz pH Scientific Trans Instrument, Singapore), Analytical Scanning Electron Microscope-Energy Dispersive X-ray (SEM-EDX, JEOL JSM-6510LA, Tokyo, Japan), Fourier-Transform Infrared Spectrometer (FTIR Perkin Elmer, Waltham, MA, USA). UV-Vis spectrometer (UV-1280, Shimadzu, Japan). Transmission Electron Microscope (TEM, Jeol 1400, Tokyo, Japan); Digital camera microscope (DGM, Motic Moticam T, Hong Kong, China).

### 2.3. Fabrication of Activated Carbon Black Nanoparticles (ACNPs)

We used the ACBMPs that had already been fabricated, according to reference [18]. However, only ACNPs produced from the activation process of using H_3_PO_4_ with a 1:1 mass ratio followed by thermal activation at 400 °C for 20 min were used. We then used the ACNPs sample after pH neutral was reached due to a series of neutralization processes employing a 0.5 M NaOH solution and washed with distilled water, which were adequately performed. Finally, we determined the particle sizes of ACNPs with a minor bit of adjustment by Tunnelling Electron Microscope (TEM), which worked according to reference [31].

### 2.4. Synthesis of Silver Nanoparticles (AgNPs) Using Aqueous-Aloe Vera Extract

Firstly, we washed about 25 g fresh aloe vera leaves of 1 cm × 1 cm in size using distilled water and chopped it in a blender machine (National, Made in Indonesia). Subsequently, we heated it with 100 mL distilled water of 90 °C, stirred at 3000 rpm for 2 h at atmospheric pressure. This mixture was incubated overnight (24 h) at room temperature and filtered using a Whatman 41 filter paper or its equivalent, held in a dark bottle until it was used and reported as a 25% *w*/*w* extract of aqueous aloe vera. After that, we diluted the extract to 5% *w*/*w* distilled water of 100 mL volume. According to reference [32] protocol, the presence of flavonoid, saponin, and tannin compounds, which are the key secondary metabolites of the aloe vera extract, were justified.

On the other hand, about 1.7 g AgNO_3_ was dissolved correctly into 50 mL distilled water in a 100 mL glass beaker. It was then gently poured in a 100 mL volumetric flask and subsequently added with distilled water up to the given mark, and it was vigorously shaken for a few seconds and kept in the dark bottle until used, with measurements of 0.1 M of 100 mL AgNO_3_. Furthermore, 10 mL aloe vera extract of 5% in concentration was mixed correctly with 10 mL AgNO_3_ of 0.1 M in concentration. We heated this mixture at 100 °C for 15 min, and let it cool at room temperature, before being agitated with ultrasonic wave (40 kHz, 2 × 50 watt) for 10 min. Finally, it was stored in a dark bottle for five days at room temperature. The developed AgNPs were then separated for 10 min by centrifugation at 5000 rpm and then dispersed again in 10 mL of distilled water and stored in a refrigerator until being used.

We justify the success of the synthesis of AgNPs by using UV-Vis’s spectrometer scanning run at the wavelength range of 250 to 700 nm. The maximum wavelength at about 430 to 500 nm was confirmed when the AgNPs formed. Besides those, the existence of flavonoid, saponin, and tannin representative compounds that stabilized the AgNPs might form core-shell nanostructure was analyzed using FTIR spectrometer equipped by germanium universal attenuated total reflectance (ATR) sample holder system. In this case, the sample of about 100 μL AgNPs was directly introduced on the ATR surface, then subsequently pressed at 3-newton force by the device analytical system and was three-time scanned at 5500 to 435 cm^−1^ default wavenumber range with resolution grade set up 4.

### 2.5. Incorporating AgNPs on ACNPs Surface

About 10 mL aqueous colloidal AgNPs produced from Section 2.4 were mixed correctly with 90 mL aqueous colloidal ACNPs produced from Section 2.3. The mixture was stirred at 3000 rpm with a magnetic stirrer for 1 h, followed by ultrasonic agitation (40 kHz, 2 × 50 watt) for 30 min. It was then centrifuged at room temperature for around 24 h (overnight), then centrifuged at 3000 rotations per minute (rpm). The collected material had been isolated from its corresponding liquid by the decantation method. The collected material was then dried using an electric furnace at 110 °C until constant in weight and heated again in an electric furnace at 500 °C under vacuum for approximately 2 h. Finally, we added 50 mL of distilled water into the material and stored it in the dark bottle until used.

### 2.6. Surface Morphology Confirmation and Particles Size Determination of ACNPs; AgNPs and AgNPs-Immobilized ACNPs

Surface morphology and particle size of ACNPs, AgNPs, and AgNPs-immobilized ACNPs materials were confirmed by using TEM operated according to reference [30] with a little bit of modification depending on the samples analyzed. In this relationship, the manufactured aqueous colloidal ACNPs, AgNPs, and AgNPs-immobilized ACNPs were each independently deposited on a TEM sample holder grid for about 100 μL. The excess liquid was extracted after about 5 min by blotting with a filter paper, allowing it to dry in an ambient environment. The samples were observed using TEM operated at 100 kV accelerating voltage.

### 2.7. Antibacterial Activity Test

About 1.5 g agarose, 0.2 g yeast extract, 0.4 g peptone, and 0.4 g NaCl were correctly mixed with 50 mL distilled water. This mixture was then heated in an Autoclave at 121 °C for 20 min. At this moment, the reaction tubes and Petri dishes, every two units, were also sterilized in the Autoclave. After that, the two reaction tubes were gently poured into about 4 mL of the liquid culture, and they were slightly tilted until the culture solidified. The rest of the culture was gently poured into the two Petri dishes with the same portion and were kept solidified at room temperature. Technically, the bacterial medium culture was prepared in a laminar cupboard under a microorganisms-free atmosphere.

On the other hand, about 0.4 g peptone, 0.2 g yeast extract, and 0.4 g NaCl were mixed correctly with 50 mL distilled water. This mixture was then sterilized in an Electrical Autoclave Sterilizer at 121 °C for 30 min. Besides those, about 1 μL of culture stock of *S. aureus* bacteria and that of *E. coli* were independently re-cultured on the already prepared solid medium enriched with nutrient agar by using an ose needle incubated at 37 °C for 24 h in a slightly tilted position. After that, both regenerated *S. aureus* and *E. coli* bacteria cultures were further cultured in the already prepared liquid medium enriched with nutrient ager using an ose needle. These cultures were then incubated at 37 °C for 24 h in a microorganism-free room.

Furthermore, about 10 mL bacterial cultures of both *S. aureus* (Gram-positive) and *E. coli* (Gram-negative) that were already prepared were independently poured into the solid medium that was also already designed. While that, several sterilized Whatman filter paper discs of 1 cm in diameter were wetted with the fabricated ACNPs, AgNPs, and AgNPs-immobilized ACNPs each of 10% *w*/*v* for filter paper disc one; filter paper disc two, and filter disc three, respectively. In addition, the freshly prepared 40 ppm antibiotic amoxicillin solution and 0.5 mL distilled water were each dropped on the other sterilized Whatman filter papers of 1 cm in diameter as a positive and negative control, respectively. Furthermore, all of the treated Whatman filter paper discs aforementioned were put carefully on the bacterial cultures previously prepared with a certain distance to each other and incubated at 37 °C for 24 h in a microorganism-free room. The antibacterial effect of the ACNPs, AgNPs, and AgNPs-immobilized ACNPs materials was represented by appearing a clear zone around the associated paper disc after incubation time. The method of the aforementioned antibacterial test performed is illustrated (see Figure S1, Supporting Information).

### 2.8. Application of the ACNPs; AgNPs, and AgNPs-Immobilized on Polyester and Cotton Fabrics

This section was conducted to evaluate whether the enhanced antibacterial properties of AgNPs, namely AgNPs-immobilized ACNPs materials, could be utilized significantly to change the basic properties of polyester and cotton fabrics from non-antibacterial to be antibacterial in properties. It was performed as follows, some pieces of both 4 × 4 cm commercially available polyester and cotton fabrics were gently soaked independently in 50 mL aqueous colloidal AgNPs-immobilized ACNPs of 1%, 5%, and 10% *w*/*v* in concentration. These soaked fabrics were then agitated with ultrasonic energy of 40 kHz, 2 × 50 watt for 30 min at 50 °C temperature. After that, the materials were taken away from the colloidal and soaked again in the same manner. Only the surface of the fabrics was reversed in position. The fabrics were taken away from the colloidal, sprayed with distilled water until the black color of the washing water disappeared, and then dried at ambient conditions. Both virgin polyester and new cotton fabrics were also treated with the same materials and procedures for comparison.

## 3. Result and Discussion

### 3.1. Synthesis of AgNPs

**Figure 1:**
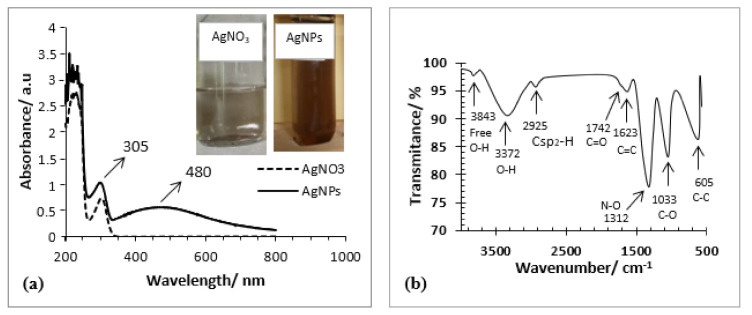
(**a**) UV-Vis spectra of silver nitrate (AgNO_3_) solution and aqueous colloidal silver nanoparticles (AgNPs); (**b**) FTIR spectra of the synthesized AgNPs.

The success of the synthesis of AgNPs from AgNO_3_ salt using aqueous aloe vera extract has been convincingly confirmed by UV-Vis’s spectrometer, as shown in Figure 1a. Meanwhile, Figure 1b shows its FTIR spectra, and Figure 1a shows appearing brown color demonstrated by the formation of the succeeding AgNPs, and in contrast, colorless AgNO_3_ solution as its precursor. It is similar to the AgNPs obtained by reference [8], which also exhibited brown color in an aqueous medium.

This phenomenon could be attributed to the correlation with the change of surface plasmon resonance (SPR) due to electron valence configuration change of Ag^+^ in AgNO_3_, i.e., [Kr] 4d^10^ (colorless) become Ag^0^ in AgNPs, i.e., [Kr] 4d^10^5s^1^ (brown color). In this case, one electron in orbital *s* (spherical orbital) with the potential energy of level five leads to AgNPs that could absorb electromagnetic waves of visible light types, which is perceived as a brown color with human eyes.

The visible light energy would cause the electron to be excited from 5*s* orbital to the next 5*p* orbital (it might be either 5*p*_x_, 5*p*_y_, or 5*p*_z_). The minimal energy gap generated between 5*s* and 5*p* orbitals is precisely equal to the quantum energy of particular photons of visible light with longer wavelengths, revealing brown color. This situation does not occur in AgNO_3_ due to no electron existing in the 5*s* orbital of the associated Ag^+^ ion so that it exhibits colorless for our naked eyes perception. This phenomenon matches with the UV-Vis spectroscopy analysis of which AgNPs provided a wavelength peak at 480 nm, while AgNO_3_ at this wavelength did not generate a rise, as shown in Figure 1. The fact was slightly different from AgNPs produced by references [3,4], who reported that the UV-Vis maximum wavelength absorption of their AgNPs obtained was 405 nm, and 402–407 nm, respectively. However, our result was closer to AgNPs produced by reference [8] and reference [33]. They reported that their AgNPs exhibited a UV-Vis maximum absorption at 420 nm and 437 nm, respectively. The differences of AgNPs UV-Vis absorption peak location (SPR spectrum) and its spectrum broadness signified the various shape and sizes of the AgNPs produced [34,35]. In this case, the longer the UV-Vis wavelength absorbed signaling, the bigger the AgNPs produced in sizes. While the broadness of the SPR spectrum relates to the shape of the AgNPs, the broader range signals more non-spherical in the formation of the AgNPs produced [33,35]. In this context, a triangle shape AgNPs of 120 nm in maximum size provided a SPR peak at about 675 nm and generated a red color, a pentagon shape AgNPs of 105 in full size provided SPR peak at about 515 nm [35].

On the other hand, FTIR spectra of the synthesized AgNPs displayed in Figure 1b are similar to those reported by several references [36,37,38,39]. Figure 1b shows seven wave number peaks ranging from 4000 cm^−1^ to 500 cm^−1^. These are 3843; 3372; 2925; 1623; 1312; 1033; and 605 cm^−1^, each reflecting a stretching vibration of free atomic bond oxygen-hydrogen (O-H); bonded atomic bond O-H; unsaturated bond carbon-hydrogen (C*sp*^2^-H); double bond carbon-carbon (C=C); single bond nitrogen-oxygen (N-O); single bond carbon-oxygen (C-O); and single bond carbon-carbon (C-C), respectively [40,41,42,43,44] (see Table S1, Supporting Information).

All of the atomic vibration types aforementioned and that displayed (see Table S1, Supporting Information) attributed that the representative molecular structures of flavonoid, saponin, or tannin shown (see Figure S2, Supporting Information) are convincingly present in the aqueous aloe vera extract. In Figure 1b, the disappearing wavenumber peaks of about 1700 cm^−1^ suggested that the functional group of carbonyls (C=O) changed to hydroxyl (OH) one. It attributed tautomeric keto-enol in the molecule of myricetin occurred. The existence of such flavonoids, tannin, and saponin can stabilize the AgNPs formed core-shell nano or microstructures of AgNPs@flovonoid-saponin-tannin. The situation remarkably matches with its UV-Vis spectra at around 200 nm wavelength peaks, displayed in Figure 1a. This wavelength was signaling that electronic transition from both non-bonding electrons (*n*) as well as *phi* (*π*) bonding ones to antibonding *phi* (π*) of the associated secondary metabolites molecular structures happened.

### 3.2. Surface Morphology

**Figure 2:**
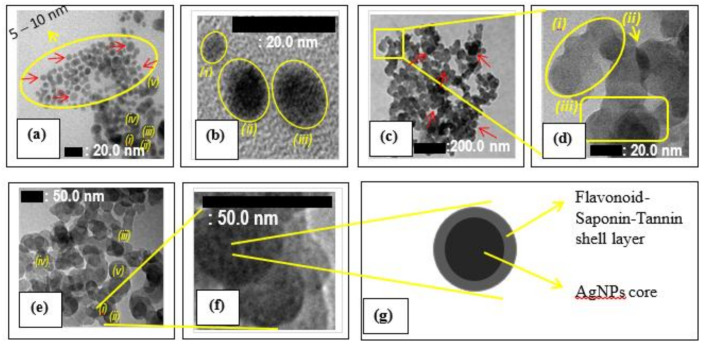
(**a**,**b**) TEM images of AgNPs; (**c**,**d**) TEM image s of ACNPs; and (**e**,**f**) TEM images of AgNPs-immobilized ACNPs; (**g**) Schematic illustration of 5–10 nm AgNPs@Flovonoid-Saponin-Tannin core-shell nanostructures immobilized on the surface of 50 nm ACNPs. Here, the particles images marked *(i)*, *(ii)*, *(iii)*, *(iv)*, and *(v)* are randomly selected particles to determined the average size of the associated particles.

Surface morphology and particle size distribution of the synthesized AgNPs, and the fabricated ACNPs explored with TEM is displayed in Figure 2. Figure 2a shows that a spherical particle cluster of about 5–10 nm in sizes (8.20 ± 2.05 nm in average size) of AgNPs could be appropriately separated from the other one of 18.80 ± 1.25 nm in average sizes when its aqueous medium was evaporated. In this case, the sizes of AgNPs particles could be approximately determined based on the associated bar marker. In addition, the standard deviation of the AgNPs particles size was calculated by employing an Excel-Microsoft Office software based on the particle’s sizes of mark *i* (20.0 nm), *ii* (20.0 nm), *iii* (19.0 nm), *iv* (17.5 nm), and *v* (17.5 nm). Meanwhile, Figure 2b shows three isolated AgNPs of which marked *i*, *ii*, *and iii* were about 5 nm, 10 nm, and 15 nm, respectively, with non-perfectly spherical shapes. This fact agrees with the UV-Vis spectrometer analysis, described in Section 3.1 in detail. Clustering the synthesized AgNPs of spherical and non-spherical shape also already happened, as shown in Figure 2a,b, respectively. The result is similar to that of many previous researchers whose publications have been reviewed in detail by reference [45].

Based on reference [45], the secondary metabolites of various plant extracts take a remarkable role in reducing silver ion (Ag^+^) to become silver metals (Ag^0^). Subsequently, the compounds assembling and stabilizing them form AgNPs with specific shapes and sizes [37,46]. It leads core-shell-like nanostructures of AgNPs secondary metabolites, which are confirmed so that the surface morphology of the synthesized AgNPs would vary depending on the typical molecular structure and composition of the associated secondary metabolites, which in our case is flavonoid, saponin, and tannin. The secondary metabolites most likely cause the synthesized AgNPs surface morphology to become rude.

On the other hand, Figure 2c,d displays the morphology and particle size distribution of the ACNPs explored with TEM. The spherical particles cluster of about 20.10 ± 1.52 in average sizes were joined to each other, some of them forming basic 2D nanostructures materials. In this case, ACNPs particles of circle mark *i* consisted of 5 particles of about 20, 20, 20, 18, and 18 nm each; that of circle mark *ii* consisted of 2 particles of about 20 and 20 nm each; and that of circle mark *iii* consisted of 3 particles of about 20, 22, and 23 nm each. Thus, by using an Excel-Microsoft Office software, the standard deviation of the ACNPs particle size was 1.52 nm. In addition, the stacking of other ACNPs particles of about 48.00 ± 8.37 nm in average size was also formed during the fabrication of ACNPs, as shown in Figure 2c marked with arrows. In contrast, some of the others include 3D nanostructure materials, as shown in Figure 2d(*i*,*iii*), respectively. Figure 2d shows other exciting facts. Adopting Lewis’s linear combination of atomic orbitals (LCAO) theory [47], this fact demonstrated that the spherical particles of around 20 nm in size could overlap each other to resemble atomic orbital overlapping shown by arrows (*ii*). It proved that such two particles’ adhesion energy is over their cohesion energy, and indicated the ACNPs particles present more on its surface rather than in its inside particles. This fact corresponded to the other research fact that observed the smaller the particles, as there were more atoms on its surface compared to its inside ones [48,49].

In conjunction to the FTIR spectra shown in Figure 1b, wavenumber peaks of the 2100 cm^−1^ to 1500 cm^−1^ range represented C=O of either carboxyl (-C=OOH), lactone (-OC=O) or ester (-C=OOR) group [18,36,38,39]. It indicated that acidic groups such as carboxyl, lactone, phenol, and basic groups such as chromene, ketones, and pyrones still exist in activated carbon produced under 600 °C heat treatment [22]. This situation causes the synthesized AgNPs to easier incorporate on the surface of the ACNPs when it has a core-shell nanostructure with such flavonoids, saponin, or tannin.

Furthermore, Figure 2e,f is TEM images of AgNPs-immobilized ACNPs. Despite AgNPs having already immobilized on the ACNPs surface, overlapping areas that connected two ACNPs to each other were still correctly occurring. It means functional groups that facilitate the connection are quite different in their properties and location. Figure 2f shows some black color dots on every ACNPs image’s surface. This phenomenon correlated to the flavonoid-saponin-tannin shell layer covering AgNPs, as shown in Figure 2g. It is attributed to the AgNPs that are chemically attached through hydrogen bonding with acidic functional groups (-COOH and -OH) present on the surface of ACNPs.

### 3.3. Antibacterial Activity Test

To evaluate the antibacterial activity of AgNPs and the influence of the ACNPs matrix on their antibacterial activity, we used *E. coli* and *S. aureus* bacteria of local strain, and the result was depicted (see Figure S3, Supporting Information). The antibacterial activity of the tested materials was evaluated according to the clear zone generated around the paper disks wetted by the associated materials tested. In this case, no clear zone generated around the distilled water-wetted paper disks (negative control), and contrary, the most expansive clear zone generated around the amoxicillin-wetted paper disks (positive control) in both *E. coli* (see Figure S3a, Supporting Information) and *S. aureus* (Figure S3b, Supporting Information). The wider the clear zone, the higher the antibacterial activity, and vice versa; the results are completely summarized in Table 1.

Using the broadest clear zone of 5.50 ± 0.00 mm in averages (positive control) on an *E. coli*-grown media as a reference to define the antibacterial properties of the materials tested, we can then propose a mathematical formula to state the antibacterial grade of the materials tested quantitively. The mathematical formula aforementioned is % α = (β ÷ γ) × 100% (antibacterial equation), where α, β, and γ each is the antibacterial activity, clear zone generated around the material tested, and the broadest clear zone generated around positive control, respectively. The antibacterial activity of amoxicillin towards *E. coli* was determined to be 100%, and that towards *S. aureus* was being 99.94% (~100%). This fact confirmed that amoxicillin is pure antibiotic medicine. However, the most important and interesting point was that the antibacterial activity of AgNPs material increased when immobilized on ACNPs, it was to *E. coli* as well as to *S. aureus* bacteria. In addition, Table 1 shows that the antibacterial activity of AgNPs increased considerably from 57.58% to 63.64% in *E. coli* (a rise of 6.06%); and that a more significant increase occurred in *S. aureus* when it was incorporated on the fabricated ACNPs materials, i.e., from 61.25% to 93.49% (increase 32.24%), and on the fabricated ACNPs materials, i.e., from 61.25% to 93.49% (increase 32.24%). It is convincing that the fabricated ACNPs can significantly increase the antibacterial activity of the synthesized AgNPs, especially towards *E. coli*, and *S. aureus* bacteria.

The fact can become a piece of additional evidence that AgNPs has a synergistic effect in antimicrobial activities, as reported by references [15,16,50]. Furthermore, the role of ACNPs in enhancing the antibacterial activity might be reasonably explained. Firstly, the AgNPs-immobilized ACNPs particles infiltrate into the bacteria cell wall and membrane, breaking down them due to the formation of hydrophilic bonding. It was facilitated by the bacterial cell wall that was commonly constructed by polymeric peptidoglycan, while the membrane has been built by various double layers phospholipids membrane [51,52,53]. The infiltration processes possibly took place since the particle sizes of AgNPs-immobilized ACNPs at just about 32.60 ± 4.88 nm in average size, based on Figure 2e,f. In this case, the standard deviation of the sizes of AgNPs-immobilized ACNPs particles determined by employing an Excel-Microsoft Office software based on the particle’s sizes mark *i* (35 nm), *ii* (30 nm), *iii* (28 nm), *iv* (30 nm), and *v* (40 nm) was about 4.88 nm. On the other hand, a bacterial cell is so much bigger than it—about more than 1000 nm. In addition, the infiltration process could be empowered by van der Waals interactions between the hydrophobic parts of the ACNPs and the bacterial cell wall and membrane.

**Figure 3:**
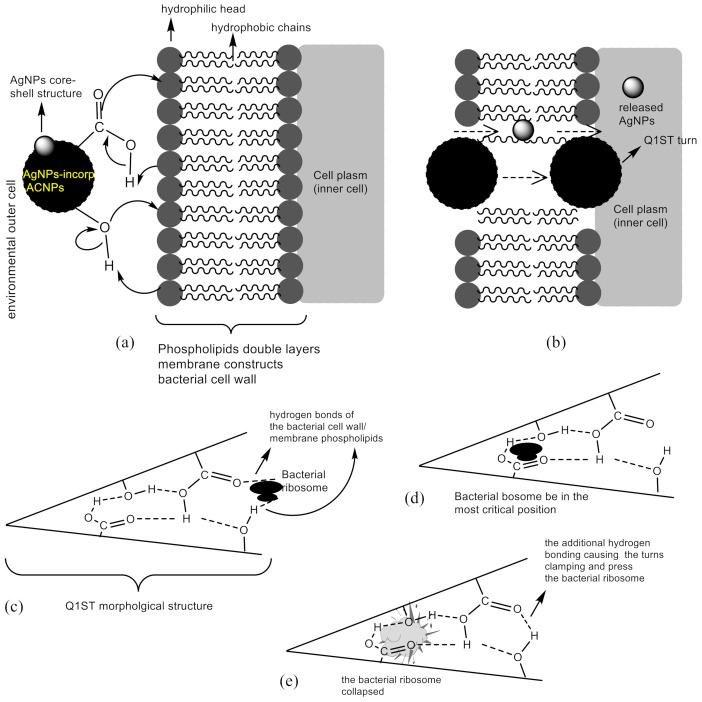
Schematic illustration of (**a**,**b**) entering process of AgNPs-immobilized ACNPs to the bacterial cell plasm; (**c**–**e**) the role of ACNPs Q1ST turn in the synergistic effect of the synthesized AgNPs.

The above actions lead to the cell wall as well as the membrane leaking and generate specific trajectories, which coincide with the AgNPs released from the ACNPs and subsequently kinetically through the cell wall and membrane entering the bacteria plasm. The entering process of AgNPs-immobilized ACNPs to the bacterial plasm aforementioned can be illustrated in Figure 3a,b. Using the surface morphology nomenclature proposed by reference [18], we used the Q1ST basin to propose the explanation of the antibacterial activity enhancement. In this explanation, certain cell organelles such as mitochondria, ribosomes, chromosomes, etc., of less than 20 nm in size were temporary deposited in the Q1ST basin generated on the ACNPs surface through hydrogen bonding, which occurred between the acidic groups and the organelles’ hydrophilic ones, e.g., ribosome, as illustrated in Figure 3c. Stretching and rocking vibrations of the hydrogen bonds then drove the bacterial ribosome to enter more deeply into the Q1ST basin until it reached the critical position, as illustrated in Figure 3d. In that position, the bacterial ribosome collapsed due to vibrational energy exposure coming from C=O, C-O, C-C, C=C, C-H, and O-H bonds which built up the Q1ST basin inner surface, including the hydrogen bonds located at the Q1ST gate, as illustrated in Figure 3e.

These processes were accompanied by releasing AgNPs from the hydrogen bonding compartments that may have existed on the surface of ACNPs. The bacteria-killing mechanism due to AgNPs itself has already been explained in detail by references [12,15,16,54,55,56].

### 3.4. Application of the Synthesized AgNPs on Textile Fabrics

**Figure 4:**
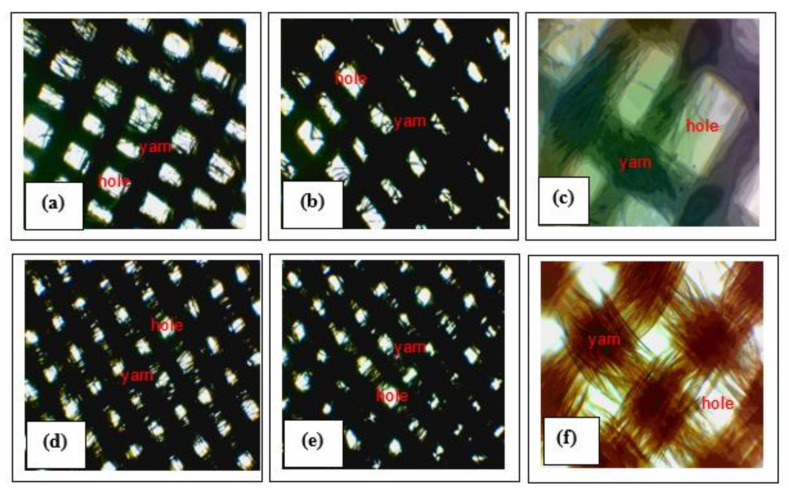
The digital camera microscope images of the cotton fabrics coated with (**a**) AgNPs-immobilized ACNPs of 5% *w*/*v* concentration, (**b**) ACNPs of 5% *w*/*v* concentration_cotton, (**c**). Original cotton fabrics; and that of the polyester fabrics coated with (**d**) AgNPs-immobilized ACNPs of 5% *w*/*v* concentration, (**e**) ACNPs of 5% *w*/*v* concentration_polyester, (**f**) Original polyester fabrics. Here, 40× magnification digital camera was performed to (**a**,**b**,**d**,**e**); and 100× magnification digital camera was performed to (**c**,**f**).

The digital camera images of the AgNPs-immobilized ACNPs materials coated on the cotton and polyester fabrics is depicted in Figure 4. Figure 4 shows that the distances generated among the cotton fabrics’ strings were broader than those in the polyester fabrics. In addition, the number of holes generated among the yarns on the cotton fabric were lesser than that on the polyester fabrics. It demonstrated that the arrangement of the polyester yarns that constructed the polyester fabrics was denser than that of the cotton yarns.

Despite the yarns’ arrangements of the cotton fabrics were less dense than that of the polyester fabrics, the cotton fibers constructed the associated threads were dumber than that of the polyester, as shown in Figure 4c,f, respectively.

The two contradictive facts aforementioned could be a unique character for the fabrics, particularly when it was coated by antibacterial material, especially AgNPs-immobilized ACNPs. In this context, the combination of the AgNPs-immobilized ACNPs and ACNPs each coated on the cotton yarns turn out to provide antibacterial properties better than that coated on the polyester yarns. The arrangement of the associated yarns is shown in Figure 4a,b,d,e respectively. In these figures we can also see that many white dots are revealed on the polyester yarn rather than on cotton. The fact demonstrates that the combination materials of AgNPs-immobilized ACNPs and ACNPs could not be adequately attached to the polyester yarn compared to the cotton yarn.

Furthermore, the antibacterial activity of both cotton and polyester fabrics after they were coated with the AgNPs-immobilized ACNPs as well as with ACNPs each were depicted (see Figure S4, Supporting Information). In this case, a clear zone has been generated around several disks of cotton and polyester fabrics wetted with amoxicillin antibiotic (positive control), AgNPs-immobilized ACNPs, and ACNPs when all were incubated on both *E. coli* and *S. aureus* bacterial cultures. In contrast, the similar disk wetted with distilled water did not a generate clear zone at all when it was positioned on the same bacterial cultures. The width generated by the aforementioned clear zones is completely summarized in Table 2.

We can see in Table 2, the cotton fabric disk wetted with amoxicillin and positioned on the *E. coli* bacterial culture generated a clear zone with the widest radius, i.e., about 10.40 mm. Based on the broadest clear zone radius then, antibacterial activity of the fabrics was calculated through the antibacterial equation, Section 3.3. By this method, the associated materials-coated textile fabrics’ antibacterial activity could be expressed in a percentage value. We have calculated that the polyester fabric coated with AgNPs-immobilized ACNPs has a higher antibacterial activity towards *E. coli* and *S. aureus* bacteria, i.e., 31.73%, and 21.15%, than the cotton fabric covered with the same materials, i.e., 19.23%, and 13.36%, respectively. The facts indicate that the density of yarn was the more critical parameter in determining the antibacterial activity of the textile fabric than the density of fibers constructed in the associated thread.

## Figures and Tables

**Table 1:** Summary of the clear zone width generated around the paper disks wetted with the materials tested regarding their antibacterial activity.

Materials, 10% in Concentration	Clear Zone Radius/mm									
	*E. coli*					*S. aureus*				
	I	II	III	Average	% Antibacterial Activity *	I	II	III	Average	% Antibacterial Activity *
AgNPs	2.50	4.50	2.50	3.17 ± 1.15	57.58	3.50	3.50	2.50	3.17 ± 0.58	61.25
AgNPs-immobilized ACNPs	3.50	3.50	3.50	3.50 ± 0.00	63.64	5.50	4.50	4.50	4.83 ± 0.50	93.49
Amoxicillin (positive control)	5.50	5.50	5.50	5.50 ± 0.00	100.00	4.50	5.50	5.50	5.17 ± 0.58	99.94
Distilled water (negative control)	0	0	0	0	0	0	0	0	0	0

* % Antibacterial activity = (Clear zone generated around the material tested ÷ The most expansive clear area developed around positive control) × 100%.

**Table 2:** Summary of the clear zone generated around the textile fabric disks, which wetted with the stated materials and positioned on *E. coli* and *S.* *aureus’s* bacterial culture.

Textile Fabrics Wetted with Materials of 5% *w*/*v* in Concentration *		Clear Zone			
		*E. coli*		*S. aureus*	
		Radius/mm	% Antibacterial Activity **	Radius/mm	% Antibacterial Activity **
Cotton	ACNPs	0	0	0	0
	AgNPs-immobilized ACNPs	2.00	19.23	1.40	13.36
	Amoxicillin (positive control)	10.40	100	8.50	81.73
	Distilled water (negative control	0	0	0	0
Polyester	ACNPs	0	0	0	0
	AgNPs-immobilized ACNPs	3.30	31.73	2.20	21.15
	Amoxicillin (positive control)	8.30	79.81	7.90	75.96
	Distilled water (negative control	0	0	0	0

* See Table S2, Supporting Information for the concentration of 1% and 10% *w*/*v*. ** % Antibacterial activity = (Clear zone generated around the material tested ÷ The most expansive clear area developed around positive control) × 100%.

## Data Availability

The relevant data of which is not displayed in this paper has been presented as Supporting Information file, and it can be accessed freely from the given link of Molecules-MDPI.

## Supplementary Materials

Schematic illustration of the antibacterial properties test of the fabricated ACNPs, AgNPs, and AgNPs-immobilized ACNPs (Figure S1); Representative molecular structure of (a) flavonoid [41], (b) saponin [42,43], and (c) tannin [44] (Figure S2); Antibacterial activity test towards (a) *E. coli*, (b) *S. aureus*. Here (1). Amoxicilin antibiotic (positive control), (2) Distilled water (negative control), (3). AgNPs-immobilized ACNPs; (4). AgNPs (Figure S3); Antibacterial activity test for cotton fabrics towards (a) *E. coli*; (b) *S. aureus*; and polyester fabrics towards (c) *E. coli*; (d) *S. aureus.* Here, (1). Wetted with amoxiciline (positive control); (2). Wetted with distilled water (negative control); (3). Wetted with AgNPs-incorporated ACNPs of 5% *w/v* in concentration; (4). Wetted with ACNPs of 5% *w/v* in concentration (Figure S4); Assignment of the synthesized AgNPs FTIR spectra, in detail (Table S1); Completely summary of the clear zone generated around the textile fabric disks, which wetted with the stated materials and positioned on *E. coli* and *S. aureus’s* bacterial culture (Table S2).

## Sample Availability

Samples of the colloidal activated carbon nanoparticles, and the synthesized colloidal silver nanoparticles are available from the authors.
