# Supplementary material for: Green Synthesized Silver Nanoparticles Immobilized on Activated Carbon Nanoparticles: Antibacterial Activity Enhancement Study and Its Application on Textiles Fabrics
Source: Molecules. 2021 Jun 22;26(13):3790. doi: 10.3390/molecules26133790 (PMC8270246; doi:10.3390/molecules26133790)
Supplement: Supplementary file 1 [file molecules-26-03790-s001.zip › molecules-1242415-supplementary.pdf]

molecules

Supplementary Material

Green Synthesized Silver Nanoparticles Immobilized on Activated Carbon Nanoparticles: Antibacterial Activity Enhancement Study and Its Application on Textiles Fabrics

Pratama Jujur Wibawa [1,*], Muhammad Nur [2], Mukhammad Asy'ari [1], Wijanarka Wijanarka [3], Heru Susanto [4], Heri Sutanto [2] and Hadi Nur [5]

[1] Department of Chemistry, Faculty of Sciences and Mathematics, Diponegoro University, Jalan Prof. H. Soedarto, SH. No.1 Tembalang, Semarang, 50275, Indonesia; asyari@live.undip.ac.id
[2] Department of Physics, Faculty of Sciences and Mathematics, Diponegoro University, Jalan Prof. H. Soedarto, SH. No.1 Tembalang, Semarang, 50275, Indonesia; mnur@lecturer.undip.ac.id (M.N.); herisutanto@live.undip.ac.id (H.S.)
[3] Department of Biology, Faculty of Sciences and Mathematics, Diponegoro University, Jalan Prof. H. Soedarto, SH. No.1 Tembalang, Semarang, 50275, Indonesia; Wijanarka@lecturer.undip.ac.id
[4] Department of Chemical Engineering, Faculty of Enginering, Diponegoro University, Jalan Prof. H. Soedarto, SH. No.1 Tembalang, Semarang, 50275, Indonesia; herususanto@lecturer.undip.ac.id
[5] Center for Sustainable Nanomaterials, Inbu Sina Institute for Scientific and Industrial Research, Universiti Teknologi Malaysia, Skudai 81310, Johor, Malaysia; and Central Laboratory of Minerals and Advanced Materials, Faculty of Mathematica and Natural Sciences, State University of Malang, Malang, 65145, Indonesia; hadinur@utm.my
* Correspondence: pratamajw@live.undip.ac.id

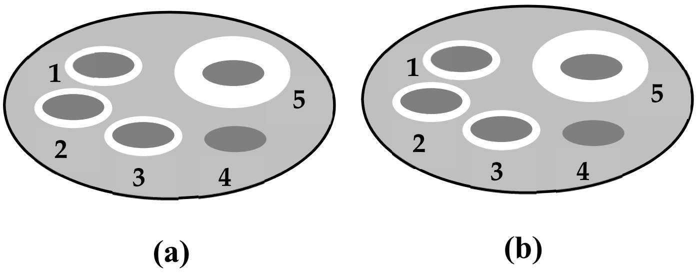

Figure S1. Schematic illustration of the antibacterial properties test of the fabricated ACNPs, AgNPs, and AgNPs-immobilized ACNPs. (**a**). Petri dish loaded with *E. coli* bacterial solid culture, (**b**). Petri dish loaded with *S. aureus* bacterial solid culture, (1). Whatman filter paper wetted with ACNPs, (2) that soaked with AgNPs, (3) flowed with AgNPs-immobilized ACNPs, (4). Whatman filter paper washed with distilled water (negative control), no clear zone generated, (5). Whatman filter paper wetted with antibiotic amoxicillin (positive control) with the broadest clear area. A clear spot with various wide was generated around samples 1, 2, and 3.

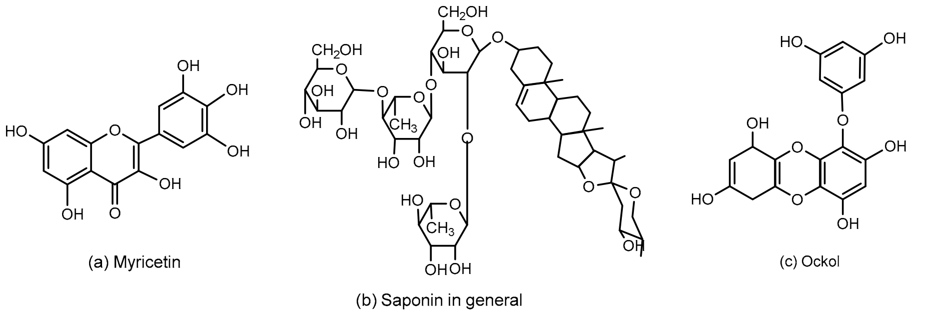

Figure S2. Representative molecular structure of (**a**) flavonoid[41], (**b**) saponin[42–43] , and (**c**) tannin[44].

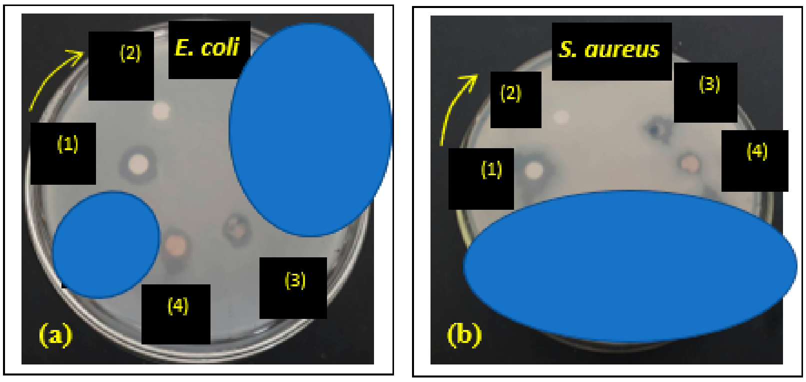

Figure S3. Antibacterial activity test towards (**a**) *E.coli,* (**b**) *S. aureus*. Here (1).Amoxicilin antibiotic (positive control), (2) Distilled water (negative control), (3).AgNPs-immobilized ACNPs; (4). AgNPs.

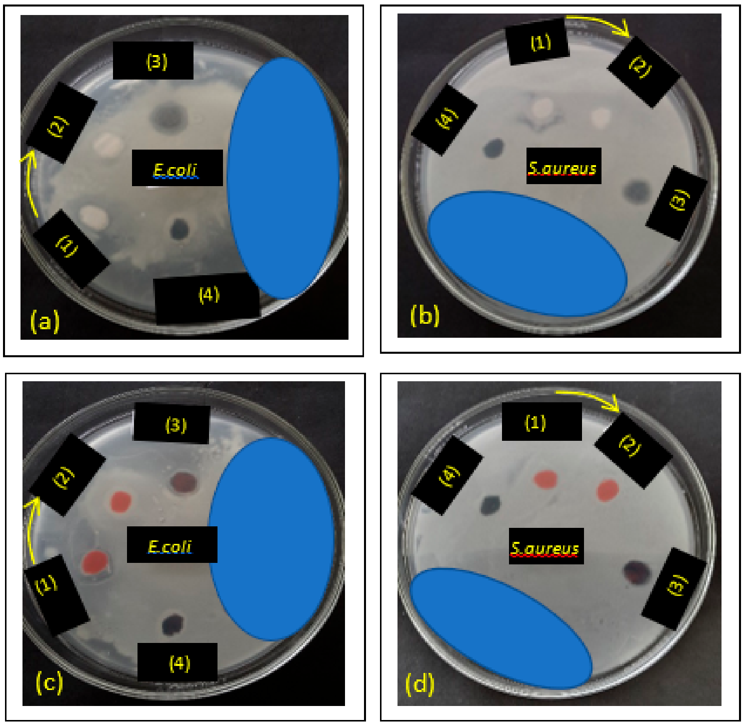

Figure S4. Antibacterial actifity test for cotton fabrics towards (**a**) *E.coli;* (**b**) *S.aureus*; and polyester fabrics towards (**c**) *E.coli;* (d) *S.aureus.* Here, (1).Wetted with amoxiciline (positive control); (2). Wetted with distilled water (negative control); (3). Wetted with AgNPs-incorporetd ACNPs of 5%w/v in cincentration; (4). Wetted with ACNPs of 5% *w*/*v* in cincentration.

Table S1. Assignment of the synthesized AgNPs FTIR spectra, in detail.

FTIR spectra		Represented Atomic Bonds Vibration	References Number
**Wavenumber Peaks/cm^{-1}**	**References**		
3843	3744–3943	free O-H stretching of water vapor	[40]
3372	3431	bonded O-H stretching of phenol	[38,39]
	3427–3436	bonded O-H stretching	[36]
	3423,15	free O-H stretching of phenol	[37]
	3420,77	free O-H stretching of alcohol	[37]
2925	2922	C-H aliphatic stretching	[38,39]
1742	1744	C=O stretching	[36,38,39]
1623	1630,32; 1631,43	C=C aromatics stretching	[37]
1312	1375	N-O stretching	[38,39]
1033	1034	C-O stretching	[36]
605	616–672, 522–575	C-C aliphatic bending	[36]
	593-630	N=O stretching	[40]

Table S2. Completely summary of the clear zone generated around the textile fabric disks, which wetted with the stated materials and positioned on *E. coli* and *S.aureus's* bacterial culture.

**Textile Fabrics Wetted With Materials of 1% *w/v* in Concentration**		**Clear Zone**			
		E. coli		***S. aureus***	
		**Radius/mm**	**% Antibacterial Activity***	**Radius/mm**	**% Antibacterial Activity ***
Cotton	ACNPs	0	0	0	0
	AgNPs-immobilized ACNPs	0	0	0	0
	Amoxicillin (positive control)	70	21	180	55.73
	Distilled water (negative control	0	0	0	0
Polyester	ACNPs	0	0	0	0
	AgNPs-immobilized ACNPs	0	0	0	0
	Amoxicillin (positive control)	323	100	204	63.16
	Distilled water (negative control	0	0	0	0

* % Antibacterial activity = (Clear zone generated around the material tested ÷ The most expansive clear area developed around positive control) × 100 %

**Textile fabrics wetted with materials of 5% w/v in concentration**		**Clear Zone**			
		E. coli		***S. aureus***	
		**Radius/mm**	**% Antibacterial Activity ***	**Radius/mm**	**% Antibacterial Activity ***
Cotton	ACNPs	0	0	0	0
	AgNPs-immobilized ACNPs	2.00	19.23	1.40	13.36
	Amoxicillin (positive control)	10.40	100	8.50	81.73
	Distilled water (negative control	0	0	0	0
Polyester	ACNPs	0	0	0	0
	AgNPs-immobilized ACNPs	3.30	31.73	2.20	21.15
	Amoxicillin (positive control)	8.30	79.81	7.90	75.96
	Distilled water (negative control	0	0	0	0

* % Antibacterial activity = (Clear zone generated around the material tested ÷ The most expansive clear area developed around positive control) × 100 %

**Textile fabrics wetted with materials of 10% w/v in concentration**		**Clear zone**			
		E. coli		***S. aureus***	
		**Radius/mm**	**% Antibacterial activity***	**Radius/mm**	**% Antibacterial activity***
Cotton	ACNPs	0	0	0	0
	AgNPs-immobilized ACNPs	0.00	0.00	0.00	0.00
	Amoxicillin (positive control)	204	63.16	323	100
	Distilled water (negative control	0	0	0	0
Polyester	ACNPs	0	0	0	0
	AgNPs-immobilized ACNPs	0.00	0.00	0.00	0.00
	Amoxicillin (positive control)	255	78.95	100	30.96
	Distilled water (negative control	0	0	0	0

* % Antibacterial activity = (Clear zone generated around the material tested ÷ The most expansive clear area developed around positive control) × 100 %
